# Strategies to reduce 28-day mortality in adult patients with bacteremia in the emergency department

**DOI:** 10.1186/s12879-024-10242-1

**Published:** 2024-12-04

**Authors:** Noémie Laurier, Angela Karellis, Xiaoqing Xue, Marc Afilalo, Karl Weiss

**Affiliations:** 1 Division of Infectious Diseases and Microbiology, Jewish General Hospital, 3755 Chemin de La Cote-Sainte-Catherine, Montreal, QC H3T 1E2 Canada; 2 McGill University, 845 Sherbrooke St W, Montreal, QC H3A 0G4 Canada; 3 Centre of Excellence in Infectious Diseases, 3755 Chemin de La Cote-Sainte-Catherine, Montreal, QC H3T 1E2 Canada; 4 Lady Davis Institute, Montreal, QC H3T 1E2 Canada; 5 Emergency Department, Jewish General Hospital, 3755 Chemin de La Cote-Sainte-Catherine, Montreal, QC H3T 1E2 Canada; 6 Université de Montréal, 2900 Edouard Montpetit Blvd, Montreal, QC H3T 1J4 Canada

**Keywords:** Bacteremia, Emergency department, Mortality, Risk factors, Management strategies

## Abstract

**Background:**

Bacteremia, a common emergency department presentation, has a high burden of mortality, cost and morbidity. We aimed to identify areas for potential improvement in emergency department bacteremia management.

**Methods:**

This retrospective cohort study included adults with bacteremia in an emergency department in 2019 and 2022. The primary outcome was 28-day mortality. Descriptive analyses evaluated demographics, comorbidities and clinical characteristics. Univariate and multivariate analyses identified mortality predictors.

**Results:**

Overall, 433 patients were included [217 males (50.1%), mean ± SD age: 74.1 ± 15.2 years]. The 28-day mortality rate was 15.2% (*n* = 66). In univariate analysis, age ≥ 70 years, arrival by ambulance, arrhythmia, congestive heart failure, recent steroid use, hypotension (< 90/60 mmHg), mechanical ventilation, cardiac arrest, intensive care unit (ICU) admission, intravenous antibiotics, pneumonia as bacteremia source, non-urinary tract infections, no infectious disease consultation, no antibiotic adjustment and no control blood cultures were significantly associated with 28-day mortality (*p* < 0.05). Malignancy showed a statistical trend (0.05 < *p* < 0.15). The above-stated sixteen variables, identified in univariate analysis, were assessed via multivariate analysis. Primarily, clinical relevance and, secondarily, statistical significance were used for multivariate model creation to prioritize pertinent variables. Five risk factors, significantly associated with mortality (*p* < 0.05), were included in the model: ICU admission [adjusted OR (95% CI): 6.03 (3.08–11.81)], pneumonia as bacteremia source [4.94 (2.62–9.32)], age ≥ 70 [3.16 (1.39–7.17)], hypotension [2.12 (1.02–4.40)], and no infectious disease consultation [2.02 (1.08–3.78)]). Surprisingly, initial antibiotic administration within 6 h, inappropriate initial antibiotic regimen and type of bacteria (Gram-negative, Gram-positive) were non-significant (*p* > 0.05).

**Conclusions:**

We identified significant mortality predictors among emergency department patients presenting with bacteremia. Referral to an infectious disease physician is the only modifiable strategy to decrease 28-day mortality with long-term effect and should be prioritized.

## Background

Bacteremia is a common presenting condition in the emergency department (ED). Recent studies have reported ED bacteremia incidence rates of up to 14% [1, 2]. Moreover, the 30-day bacteremia mortality rate in Canada is 17.0% [3]. Due to its high incidence and mortality burden, bacteremia also exerts an important economic stress on many stakeholders, including patients, healthcare systems and governments. In England, the 2019 economic burden of *Escherichia Coli* bacteremia in secondary health centers was reported to be approximately 18 million US dollars [4].

To reduce bacteremia-associated burden, the assessment of risk factors will allow healthcare professionals to ascertain patients’ individualized risk, to appropriately manage care. ED bacteremia mortality risk factors can include liver, respiratory, cardiovascular and neurological dysfunction, malignancy, anemia, septic shock, need for organ support and polymicrobial infections [5]. Additional studies have shown an increased risk of adverse outcomes, including urgent hospitalization and return to ED, among bacteremia patients who received untimely and inappropriate antibiotics [6–11].

Due to the lacking published data regarding ED bacteremia mortality, our goal was to identify areas of potential improvement in ED bacteremia management and the populations most at risk of mortality, to enable strategic allocation of healthcare resources.

## Methods

### Study design and setting

This retrospective cohort review study was conducted at the Jewish General Hospital in Montreal, Quebec, using data between January 2019 and December 2019 and between January 2022 and December 2022. It included ED adult patients with a significant positive blood culture. Blood cultures were ordered when clinically indicated by the ED staff; signs and symptoms of an active infection may have included fever, hypothermia, leukocytosis, hypotension (< 90/60 mmHg) with or without tachycardia, and any other signs suggestive of sepsis. Blood cultures were also ordered in cases of suspected pyelonephritis, meningitis, endocarditis, cholangitis, catheter-related infections, and other severe infections. Following confirmation of the bacteremia, control blood cultures were ordered at the discretion of the attending physician to confirm absence of infection following antimicrobial therapy. This tertiary-care center has the busiest ED in Quebec with approximately 100,000 visits per year. The years 2020 and 2021 were excluded because of the management impacts caused by the Covid-19 pandemic. Ethics review was not necessary since this retrospective study was conducted in the context of a quality assurance program. However, we can attest that this study is in accordance with the ethical principles of the Declaration of Helsinki [12].

### Patient population

Adult patients (≥ 18 years) with a positive blood culture were eligible for inclusion. Positive blood cultures obtained in the same two-week period were counted as a single episode of bacteremia (*n* = 629 duplicates). Patients with known blood contaminants, those who never received antibiotics and patients discharged on palliative care were excluded. Pathogens were considered contaminants when they represented common skin flora (e.g. coagulase-negative staphylococci), particularly if only present in a single bottle. An infectious disease (ID) physician reviewed all pathogens on a case-by-case basis to confirm inclusion of all significant positive blood cultures.

### Key outcome measures and data collection

Data from index ED admission was collected in a confidential Excel spreadsheet. Bacteremia is defined by the Centers for Disease Control as a positive blood culture with viable bacteria in the patient’s bloodstream [13]. The primary outcome was 28-day all-cause mortality. Our secondary outcome was 7-day all-cause mortality.

Patient parameters to collect were established during study design and prior to data collection. The following data were extracted from electronic medical records: age, sex, arrival mode, recall to hospital (return to ED after initial hospital discharge due to receipt of positive blood culture results), 7-day and 28-day mortality. Comorbidities, recent steroid use (< 30 days), recent hospitalization, vital signs, source of infection, ED antibiotic administration characteristics, control blood cultures requests and ID physicians’ management were assessed. As antimicrobials were administered prior to receipt of microbiological analysis results, empirical antibiotic treatment was provided as first-line therapy. These were defined as “appropriate” if the bacteria was considered susceptible in-vitro to the prescribed antibiotic. When appropriate, the spectrum of antibiotics was narrowed when susceptibility results were obtained from the laboratory. Timepoints included ED admission, ward admission, hospital discharge, first physician assessment in ED, first antibiotic administration and ID consultation.

### Data analysis

Descriptive statistics, including means (standard deviations), medians (interquartile ranges) and proportions, were used to describe demographics, comorbidities and mortality of the study population. Univariate analyses including Student’s T-test/Mann–Whitney for continuous variables and chi-square/Fisher’s exact tests for categorical variables were used to study the relationship between each variable alone with 28-day mortality. Clinically significant variables with *p* < 0.15 in univariate chi-square analysis were included in the multivariate logistic regression model. When variables fell into similar categories, one representative variable per group was selected and only those deemed most clinically important were chosen for the multiple variable analysis. Results are presented as odds ratios (ORs) with 95% confidence intervals (CIs). All analyses were conducted using SAS statistical analysis software (SAS V 9.4) (SAS Institute, Cary, NC, USA).

## Results

### Study population

Overall, 433 patients were included in this study, of which 49.9% were females and 50.1% were males. All known blood contaminants (*n* = 583), patients who never received antibiotics (*n* = 3) and patients discharged on palliative care (*n* = 5) were excluded from our study sample. The mean ± SD age of the cohort was 74.1 ± 15.2 years. The 7-day and 28-day all-cause mortality rates were 7.4% and 15.2%, respectively. Overall, 59.6% of patients had a bacteremia caused by Gram-negative bacteria. Hypertension (65.6%), type 2 diabetes (30.5%), malignancy (29.6%) and arrhythmia (23.1%) were the most common comorbidities. Also, control blood cultures were ordered for 95.8% of patients, 91.9% of patients had an appropriate initial antibiotic therapy for which the pathogen was sensitive to the prescribed medication, and 76.0% of patients had an antibiotic adjustment following microbiology results (Table 1).

**Table 1:** Patient and infection characteristics

Variable	*N* = 433	
Age, years, mean (SD)	74.1 (15.2)	
Sex, n (%)		
Male	217 (50.1)	
Female	216 (49.9)	
Mortality, n (%)		
7-day mortality	32 (7.4)	
28-day mortality	66 (15.2)	
Recall rate^a^, n (%)	72 (16.6)	
Comorbidity, n (%)		
Hypertension	284 (65.6)	
Arrhythmia	100 (23.1)	
Congestive heart failure	54 (12.5)	
Coronary heart disease	79 (18.2)	
Chronic lung disease	76 (17.6)	
Diabetes type 2	132 (30.5)	
Liver cirrhosis	11 (2.5)	
Chronic kidney disease stage 4 or less (eGFR < 30 mL/m/1.73 m^2^)	15 (3.5)	
Rheumatic disease	50 (11.5)	
Transplantation	3 (0.7)	
Malignancy	128 (29.6)	
Source of infection, n (%)		
Urinary tract infection	150 (34.6)	
Pneumonia	79 (18.2)	
Abdominal	71 (16.4)	
Skin and soft tissue	35 (8.1)	
Vascular	31 (7.2)	
Bone	23 (5.3)	
Central nervous system	13 (3.0)	
Surgical	11 (2.5)	
Ear-nose-throat	5 (1.2)	
Catheter	3 (0.7)	
Ophthalmological	1 (0.2)	
Undetermined source	9 (2.1)	
Febrile neutropenia	2 (0.5)	
Type of bacteria, n (%)		
Gram-positive	171 (39.5)	
Gram-negative	258 (59.6)	
ID consultation, n (%)	237 (54.7)	
Consultation under 24 h	87 (36.7)	
Consultation between 24 h-72 h	88 (37.1)	
Consultation between 72 h-7 d	49 (20.7)	
Consultation over 7 d	13 (5.5)	
Control blood culture ordered, n (%)	415 (95.8)	
Appropriate initial antibiotic, n (%)	398 (91.9)	
Antibiotic adjustment, n (%)	329 (76.0)	
Time-related outcomes from index ED admission and physician assessment to different endpoints	Median (IQR)	Mean (SD)
ED admission; Physician assessment (h)	1.1 (0.4–2.0)	1.5 (3.3)
ED admission; First antibiotic administration (h)	4.8 (2.1–12.2)	10.4 (15.3)
ED admission; Hospital discharge (d)	9.5 (4.8–19.7)	16.2 (23.7)
Physician assessment; First antibiotic administration (h)	3.3 (0.9–10.0)	8.8 (15.0)

^a^Patients who were initially discharged from the ED and were called back following receipt of positive blood culture results

The mean ± SD time from admission to first physician assessment was 1.5 ± 3.3 h and to first antibiotic administration 10.4 ± 15.3 h. Of note, 40/433 (9.2%) patients received their antibiotics more than 24 h following ED admission, of which 15 were recall patients who seemed initially well. This minority of 40 outlier patients inflated the mean time to antibiotic administration. Also, following physician assessment in ED, it took a mean ± SD of 8.8 ± 15.0 h for first antibiotic administration. Patients admitted with bacteremia in ED were in hospital for a mean ± SD of 16.2 ± 23.7 days. Additionally, 16.6% of patients were recalled following hospital discharge due to new positive blood cultures or need for treatment modification. Finally, 237 (54.7%) patients had an ID consultation, out of which 87 (36.7%) were seen in under 24 h, 88 (37.1%) between 24–72 h, 49 (20.7%) between 72 h and 7 days and only 13 (5.5%) over 7 days (Table 1). The most common bacteria were *E. coli* (33.9%), *Staphylococcus aureus* (MSSA) (12.2%), *Klebsiella pneumonia*e (11.5%) and *Proteus mirabilis* (5.8%) (Table 2).

**Table 2:** Pathogen categories present among study participants with monomicrobial and polymicrobial bacteremia in the ED

Pathogen Category	Included Bacteria	Frequency, n (%)^a^
*Escherichia coli*	*Escherichia coli*	147 (33.9)
*Staphylococcus* species		
Methicillin-sensitive *Staphylococcus aureus*	Methicillin-sensitive *Staphylococcus aureus*	53 (12.2)
Coagulase-negative *Staphylococci*	*Staphylococcus epidermidis, Staphylococcus haemolyticus, Staphylococcus lugdunensis, Staphylococcus pattenkoferi*	7 (1.6)
Methicillin-Resistant *Staphylococcus aureus*	Methicillin-Resistant *Staphylococcus aureus*	3 (0.7)
*Klebsiella pneumoniae*	*Klebsiella pneumoniae*	50 (11.5)
*Streptococcus* species		94 (21.7)
*Streptococcus pneumoniae*	*Streptococcus pneumoniae*	23 (5.3)
Group B* Streptococcus*	*Streptococcus agalactiae*	14 (3.2)
Group G* Streptococcus*	*Streptococcus dysgalactiae*	12 (2.8)
Group A* Streptococcus*	*Streptococcus pyogenes*	8 (1.8)
*Streptococcus gallolyticus*	*Streptococcus gallolyticus [pasteurianus]*	6 (1.4)
Other *Streptococcus* species	*Streptococcus anginosus, Streptococcus constellatus, Streptococcus viridans, Streptococcus mitis/oralis, Streptococcus parasanguinis, Streptococcus pasteurianus*	31 (7.2)
*Proteus mirabilis*	*Proteus mirabilis*	25 (5.8)
*Enterococcus faecalis*	*Enterococcus faecalis*	22 (5.1)
*Pseudomonas aeruginosa*	*Pseudomonas aeruginosa*	10 (2.3)
*Bacteroides* species	*Bacteroides caccae, Bacteroides fragilis, Bacteroides ovatus, Bacteroides thetaiotaomicron*	8 (1.8)
*Salmonella* species	*Salmonella dublin, Salmonella enterica, Salmonella typhi*	8 (1.8)
*Serratia marcescens*	*Serratia marcescens*	7 (1.6)
*Morganella morganii*	*Morganella morganii*	5 (1.2)
*Acinetobacter* species	*Acinetobacter baumanii, Acinetobacter radioresistens, Acinetobacter pittii*	4 (0.9)
*Enterobacter cloacae*	*Enterobacter cloacae*	4 (0.9)

^a^As certain patients had polymicrobial infections, these frequencies will not sum to 433. The denominator used for all percentages remains the total number of patients in the cohort: 433

### Univariate analysis

Univariate chi-square analysis yielded 15 significant associations with mortality (*p* < 0.05) and one statistical trend (0.05 < *p* < 0.15) (Table 3). These include: age ≥ 70 years (mortality rate among patients without vs. with risk factor: 6.4% vs. 19.5%), arrival by ambulance (3.9% vs. 21.5%), arrhythmia (12.3% vs. 25.0%), congestive heart failure (CHF) (13.5% vs. 27.8%), malignancy (13.1% vs. 20.3%), recent steroid use (13.4% vs. 24.3%), hypotension (12.4% vs. 32.8%), mechanical ventilation (11.5% vs. 24.2%), cardiac arrest (12.4% vs. 75.0%), ICU admission (10.3% vs. 43.1%), and intravenous (IV) antibiotics (2.3% vs. 28.2%). Pneumonia as bacteremia source (9.6% vs. 40.%), infection other than urinary tract infection (UTI) (2.7% vs. 21.9%), the absence of an ID consultation (10.6% vs. 20.9%), absence of antibiotic adjustment (6.4% vs. 43.3%) and absence of control blood cultures (13.3% vs. 61.1%) were also key mortality determinants. A significantly higher proportion of patients with primary pneumonia died (40.5%) compared to patients with alternate primary sources (9.6%). Although initial antibiotic administration at arrival (≤ 6 h vs. > 6 h), inappropriate initial antibiotic regimen (based on antibiotic susceptibility profiles) and type of bacteria (Gram-positive vs. Gram-negative) did not achieve the pre-established statistical trend threshold (*p* < 0.15), as past studies had previously identified these as significant mortality risk factors, we evaluated them in an ad hoc multivariate analysis. This ad hoc analysis was performed to confirm their non-significance on 28-day mortality, which was the case as these three variables were found to be non-significant.

**Table 3:** Risk factor assessment for 28-day mortality of 433 adults with bacteremia in the ED

Risk factor, n (%)	28-Day Mortality		*p*-value
	**Without risk factor [n/N (%)]**	**With risk factor [n/N (%)]**	
Age ≥ 70 years	9/141 (6.4)	57/292 (19.5)	0.0003^a^
Sex, female	28/217 (12.9)	38/216 (17.6)	0.1837
Ambulance	6/154 (3.9)	60/279 (21.5)	< 0.0001^a^
Comorbidity			
Hypertension	20/149 (13.4)	46/284 (16.2)	0.4843
Arrhythmia	41/333 (12.3)	25/100 (25.0)	0.0039^a^
Congestive heart failure	51/379 (13.5)	15/54 (27.8)	0.0134^a^
Coronary heart disease	51/354 (14.4)	15/79 (19.0)	0.3024
Chronic lung disease	50/357 (14.0)	16/76 (21.1)	0.1580
Diabetes type 2	41/301 (13.6)	25/132 (18.9)	0.1907
Liver cirrhosis	64/422 (15.2)	2/11 (18.2)	0.6778
Chronic kidney disease stage 4 or less (eGFR < 30 mL/m/1.73 m^2^)	62/418 (14.8)	4/15 (26.7)	0.2617
Rheumatic disease	57/383 (14.9)	9/50 (18.0)	0.5343
Transplantation	66/430 (15.4)	0/3 (0.0)	> 0.9999
Malignancy	40/305 (13.1)	26/128 (20.3)	0.0778^b^
Medication use within 14 days before ED admission			
NSAID	33/216 (15.3)	31/210 (14.8)	0.8930
Steroid	48/359 (13.4)	18/74 (24.3)	0.0213^a^
Systemic antibiotic	46/315 (14.6)	18/111 (16.2)	0.7574
Medical history before ED admission			
Central venous catheter placed within 14 d	62/413 (15.0)	4/19 (21.1)	0.5104
Dialysis within 30 d	65/422 (15.4)	1/10 (10.0)	> 0.9999
Hospitalization within 90 d	25/194 (12.9)	41/238 (17.2)	0.2285
Medical implant placed within 90 d	64/406 (15.8)	2/26 (7.7)	0.4000
Body temperature ≤ 36.0 °C or ≥ 39.0 °C	49/336 (14.6)	16/96 (16.7)	0.6284
Hypotension (< 90/60 mmHg)	46/372 (12.4)	20/61 (32.8)	0.0002^a^
Mechanical ventilation	35/305 (11.5)	31/128 (24.2)	0.0012^a^
Cardiac arrest	51/413 (12.4)	15/20 (75.0)	< 0.0001^a^
Admission to intensive care unit	38/368 (10.3)	28/65 (43.1)	< 0.0001^a^
Infectious disease characteristics			
Pneumonia	34/354 (9.6)	32/79 (40.5)	< 0.0001^a^
Source other than urinary tract infection	4/150 (2.7)	62/283 (21.9)	< 0.0001^a^
Gram-negative bacteria	27/171 (15.8)	37/258 (14.3)	0.8056
Absence of infectious disease consultation	25/237 (10.6)	41/196 (20.9)	0.0031^a^
Absence of control blood culture	55/415 (13.3)	11/18 (61.1)	< 0.0001^a^
Antibiotic therapy during bacteremia			
Inappropriate initial antibiotic	61/398 (15.3)	5/35 (14.3)	> 0.9999
Intravenous antibiotic	5/217 (2.3)	61/216 (28.2)	< 0.0001^a^
Absence of adjustment of antibiotic	21/329 (6.4)	45/104 (43.3)	< 0.0001^a^
Delay to antibiotic administration > 6 h	38/251 (15.1)	28/182 (15.4)	> 0.9999

^a^Denotes statitiscal significance (*p* < 0.05)

^b^Denotes statistical trend (0.05 < *p* < 0.15)

### Multivariate logistic regression

The above sixteen variables identified in univariate analysis (15 significant associations and 1 statistical trend) were evaluated in multivariate analysis. However, to avoid a compounded effect, when variables led to similar outcomes, one in each group was selected as a representative variable. In the group of ambulance and ICU admission, with patients being very vulnerable in both cases, ICU admission was retained. Among hypotension, mechanical ventilation, IV antibiotic administration, CHF, arrhythmia, malignancy and cardiac arrest, hypotension was retained. Among absence of an ID consultation, absence of antibiotic adjustment and absence of control blood cultures, the former was retained in the model. This was performed in the multivariate logistic regression analysis to ensure that redundancy was avoided in the assessment of 28-day mortality. As such, the risk factors that were retained in the model were: age, hypotension, pneumonia, absence of ID consultation and ICU admission (Table 4). The multivariate analysis identified ICU admission as the strongest risk factor of 28-day mortality [adjusted OR (95% CI): 6.03 (3.08–11.81)], followed by pneumonia as the bacteremia source [4.94 (2.62–9.32)], age ≥ 70 [3.16 (1.39–7.17)], hypotension [2.18 (1.02–4.40)], and absence of ID consultation [2.02 (1.08–3.78)]. This model’s Hosmer–Lemeshow Goodness-of-Fit Test *p*-value was 0.2449.

**Table 4:** Multivariate logistic regression model of risk factors for 28-day mortality of ED bacteremia

Variable	Odds Ratio (95% Confidence Interval)	*p*-value
Age ≥ 70 years	3.16 (1.39–7.17)	0.0060
Hypotension	2.12 (1.02–4.40)	0.0444
Pneumonia	4.94 (2.62–9.32)	< 0.0001
Absence ID consultation	2.02 (1.08–3.78)	0.0275
ICU admission	6.03 (3.08–11.81)	< 0.0001

## Discussion

This retrospective study evaluated patients’ demographics and comorbidities, the impact of an ID consultation and bacteremia primary source, aiming to identify strategies to decrease bacteremia mortality. Our multivariate model identified five key variables associated with increased mortality risk. Three were non-modifiable: age ≥ 70 years, ICU admission and pneumonia as the bacteremia source; one, hypotension at admission, must be punctually corrected, but only one was potentially modifiable in the long-term effect: an ID consultation.

Our 28-day mortality rate was 15.2%, which aligns with the 5–17% range reported in the literature for patients presenting to the ED with bacteremia [2, 3, 5, 14–16]. The mean age of participants was older than past studies, which can explain the mortality rate closer to the upper limit of published estimates [17]. Moreover, our 7-day mortality rate of 7.4% is lower than the previously documented 8.4% rate in China [2]. Although bacteremia is less discussed in mainstream medical news than other illnesses, bacteremia is a very severe condition in terms of global outcome, and its mortality rate in our study and in previously published studies [2, 3, 5, 14–16] is higher when compared to the 28-day mortality rate of acute myocardial infarction (MI). Indeed, a recent retrospective study with 5,900 patients reported a MI mortality rate of 6.3% [18], while a global meta-analysis of approximately three million patients showed MI mortality rates of 3.8–9.5% [19]. Yet, substantially more research examines actions to modify the outcomes in MI management than with bacteremia in the ED.

**Fig. 1:**
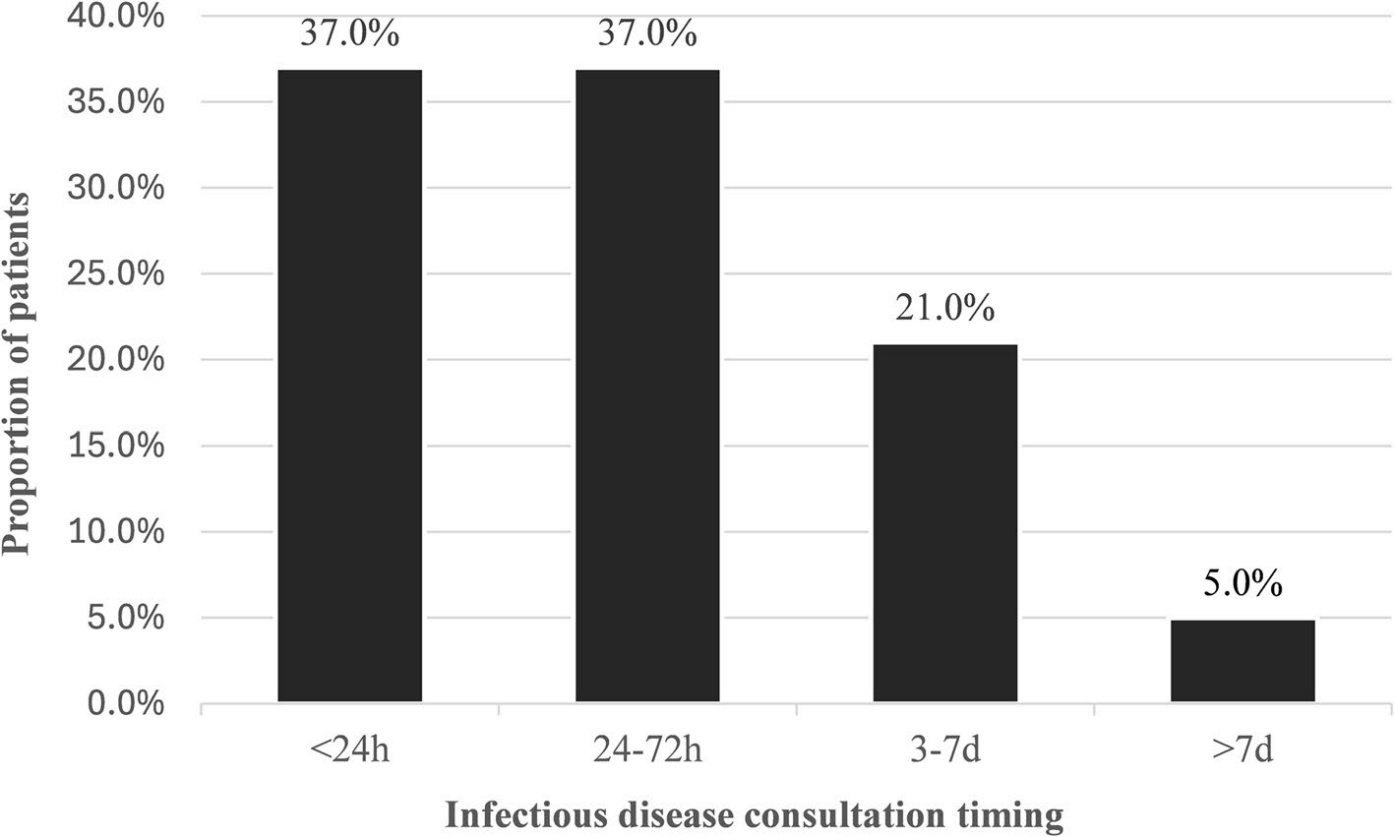
ID consultation timings distribution among the 237 patients who received an ID consultation

ID consultation following bacteremia diagnosis was identified as the only modifiable strategy to significantly decrease 28-day mortality by two-fold. This is the first study to report this outcome in a cohort consisting of only ED patients. A previous cohort study including 400 bacteremia hospital episodes, unrestricted to ED, highlighted that ID consultation on the day of positive blood culture results decreased mortality by 70%, while reducing the duration of broad-spectrum antibiotic therapy and lowering antimicrobial treatment costs [20]. Another study evaluating only *Staphylococcus aureus* bacteremia among 892 patients found a three-times lower mortality rate among patients who received ID consultations (11.6% vs. 34.6%, *p* < 0.001) [21]. The clinical expertise of ID physicians plays a crucial role to identify and control the infection source, as well as to manage the best antibiotic therapy [22]. Importantly, in our study, 73.8% of ID consultations were done within the first 72 h following ED arrival and 94.8% of all ID consultations were completed within 7 days (Fig. 1), which underlines the impact of an early demand for this kind of expertise and reinforces our main finding that ID consultations have a direct impact on 28-day mortality.

Our study identified age as a significant predictor of all-cause mortality among patients with bacteremia. Published literature concords with this finding as age is a risk factor for several health conditions, including bacteremia [17]. In fact, immunosenescence associated with age puts the elderly at greater risk of presenting to the ED with atypical bacteremia features, such as the absence of fever, chills, or hypotension [23, 24]. These atypical bacteremia characteristics may impede timely diagnosis in patients already weakened by their overall frailty. It may explain why patients ≥ 70 years were three times more likely to undergo fatal consequences in our study.

Additionally, ICU admission represents the greatest risk factor of 28-day mortality in the present study [adjusted OR (95% CI): 6.03 (3.08–11.81)]. This finding is not surprising considering that some of the main indications for ICU admission are hemodynamic compromise, respiratory failure, intubation and sepsis [25]. Infection source was also found to impact mortality. Patients with primary pneumonia were nearly five times more likely to die than those with infections originating from elsewhere. This may be explained by the fact that patients with pneumonia-caused bacteremia are more vulnerable and have more severe disease than patients with other infection sources [26]. Moreover, hypotension at presentation put our cohort approximately two-times more at risk of mortality than non-hypotensive patients. Unfortunately, we did not explicitly evaluate sepsis in our analysis, but hypotension is a main clinical feature of sepsis [27]. We hypothesize some of the hypotensive patients may have experienced a septic shock, which may explain the importance of hypotension among bacteremic patients [28].

**Fig. 2:**
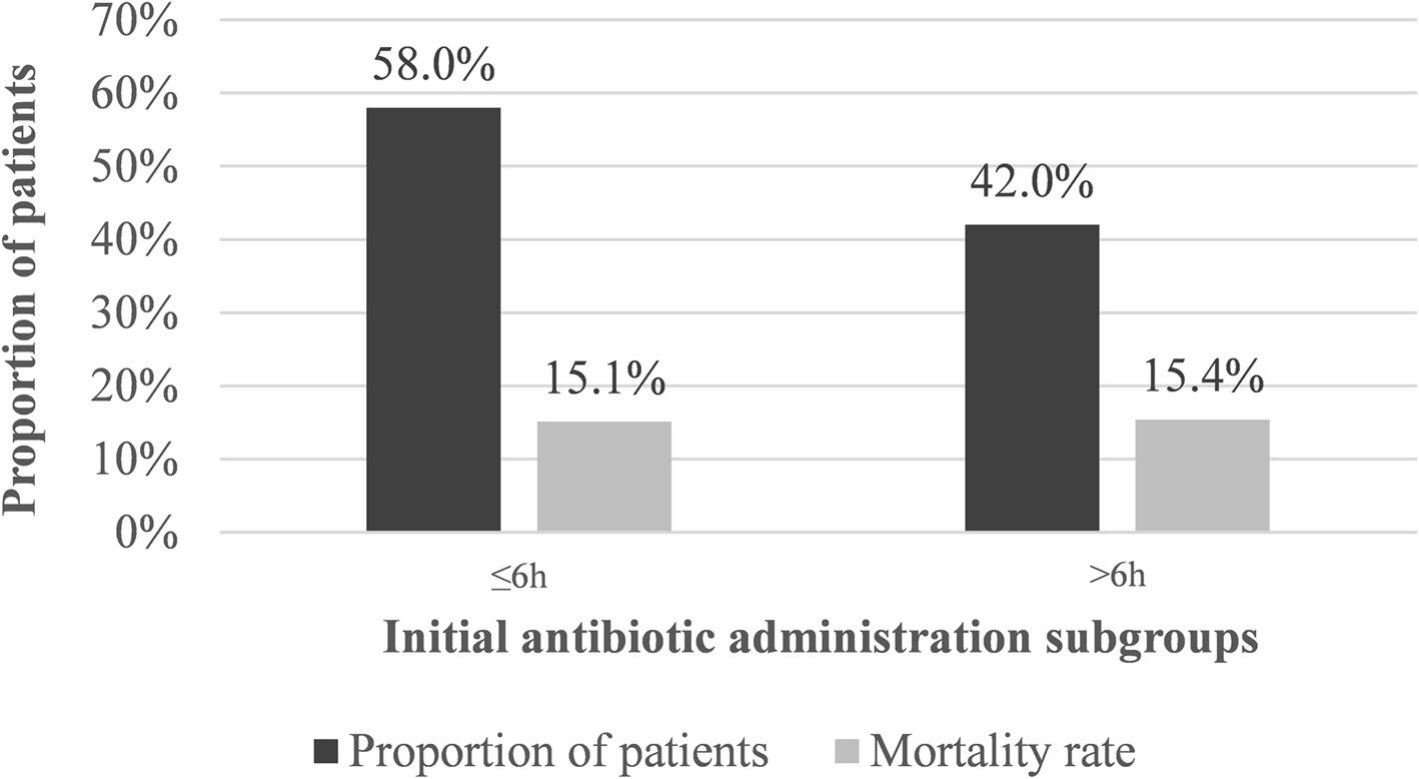
Proportion of patients and mortality rates in both initial antibiotic administration subgroups (≤ 6 h vs. > 6 h). Mean timing of initial antibiotic administration ≤ 6 h: 2.96 h. Mean timing of initial antibiotic administration > 6 h: 20.58 h

Furthermore, as opposed to past studies [29–31], antibiotic administration within 6 h of ED admission did not impact 28-day mortality within our study population (Fig. 2). This was an unexpected result given a meta-analysis of 15 cohort studies noted a heightened mortality risk for every additional hour of antibiotic delay (OR: 1.07; 95% CI: 1.06–1.08; *p* < 0.0001) [11]. Two administration subgroups may explain why antibiotic timing was not significantly associated with 28-day mortality in this cohort. According to their clinical, biological and medical conditions, patients who received an initial antibiotic within 6 h were less stable than patients who received it later. Indeed, a higher proportion of patients who received antibiotic administration within 6 h arrived by ambulance (comparing proportion of patients with risk factor who received initial antibiotic administration ≤ 6 h vs. > 6 h: 70.9% vs. 55.4%), had temperature of ≤ 36 °C or ≥ 39 °C (29.5% vs. 12.1%), hypotension (17.9% vs. 3.3%), mechanical ventilation (41.8% vs. 12.6%) and cardiac arrest (5.6% vs. 3.3%). The same is true for comorbidities: cancer (33.5% vs. 24.2%), arrhythmia (25.5% vs. 19.8%), CHF (14.3% vs. 9.9%) and diabetes type 2 (33.5% vs. 26.4%). Hence, the non-significance of this variable should be interpreted cautiously after understanding that most vulnerable patients received timely antibacterial therapy.

Unexpectedly, our study did not show a significant association between antibiotic appropriateness and mortality, as opposed to published literature. A previous retrospective cohort study reported a higher mortality rate among patients who received inappropriate initial antibiotic administration followed by antibiotic adjustment (25.8%) when compared to those with appropriate first antibiotic administration (10.5%) [7]. The non-significance in our study may be due to the low sample size of patients who received an inappropriate initial antibiotic (35/433, 8.1%), for which the pathogen was resistant to the first antibiotic administered. Emergency physicians prescribed a first dose of very broad-spectrum antibiotics as an initial treatment due to the severity of the patient’s presenting condition. Moreover, most patients within the inappropriate initial antibiotic subgroup received a second appropriate antibiotic rapidly, to which the pathogen was sensitive to, to fight the infection. Indeed, 31 patients (31/35, 88.6%) from this subgroup had an antibiotic adjustment. Unfortunately, the appropriateness of the second antibiotic for these 31 cases was not evaluated. Antibiotic appropriateness should remain a primary focus in management strategies to reduce ED bacteremia mortality. To decrease risks of infection caused by multi-drug resistant organisms while minimizing risks of bacteremia complications, the recommended antibiotic selection strategy aims to prioritize identifying the focus of infection and treating accordingly. Patient-related risk factors and whether the bacteremia was community or hospital acquired should also be taken into consideration during antibiotic selection [32]. However, if the source of infection is not identifiable and/or if the patient is unstable, trying to restrict the initial spectrum of the prescribed antibiotic should not be a priority in an ED setting. Most importantly, emphasis must be put on adjusting for narrower spectrum antibiotics when the bacteria have been identified and sensitivity results are available.

Finally, the distribution of bacteremia causative pathogens at our ED follows usual trends. *E. coli*,* S. aureus*, *K. pneumoniae* and *P. mirabilis* were among the most common pathogens [33, 34]. Gram-negative bacteria were only 7% more prevalent than Gram-positive bacteria, and previous studies have reported Gram-negative infections to be more lethal than Gram-positive ones [35]. However, the type of bacteria (Gram-negative vs. Gram-positive) did not significantly impact mortality in our study (*p* = 0.8056). The most frequent source of infection in our analysis was UTI, which is the most common source of community-acquired bacteremia [34]. More than 90% of UTI cases are due to Gram-negative enteric bacteria, notably *E. coli* [36]. Conversely, pneumonia can be caused by both Gram-negative and positive pathogens [37, 38]. Thus, we hypothesize that mortality results from Gram-negative bacteria were diluted by the high frequency of Gram-negative UTI infections in our cohort, even if they tend to cause more severe infections when originating from other sources. Overall, the source of infection should be prioritized over the type of bacteria (Gram-negative vs. Gram-positive) when evaluating bacteremia mortality risk in ED.

Our study findings can lead to multiple implications on individual, hospital and societal levels. Healthcare staff have been overburdened, particularly in Canada’s EDs [39]. While initial assessment of each patient’s condition represents an important task for all ED physicians, consultations to ID experts may help alleviate their workload and save time in patient management, particularly as our analysis has highlighted that ID consultations promote survival. However, it is important to note that ID consultations may not be feasible in all centers without positive blood cultures. Therefore, each facility is to individualize their management strategy per their specific center’s available resources. Additionally, our analysis has identified the patients with bacteremia who are most predisposed to mortality, such as the elderly (≥ 70 years), those diagnosed with primary pneumonia, hypotensive and ICU patients. We recommend allocating extra resources to these individuals following ED diagnosis to maximize benefits.

Our retrospective study is a single-center study which limits the generalizability of its results, particularly to specific high-risk populations (e.g., transplant patients). However, the Jewish General Hospital has the busiest ED in the province of Quebec and is a large tertiary-care university center with a wide array of patients across specialties. We were also limited in our study, as charts were sometimes deemed incomplete. While missing data can represent an important issue in chart review studies, the sole variable that was importantly affected by missing data was the Glasgow Coma Scale (unavailable for 71.8% of patients), which prevented its evaluation as a risk factor of mortality in our study, as well as the calculation of the Pitt-Bacteremia Score [40], the Bloodstream Infection Mortality Risk Score [41] and the Sepsis-related Organ Failure Assesment Score [42]. We also excluded the Covid-19 pandemic years (2020/2021), as these are outlier years in terms of standard medical practice [43]. The inclusion of patients from both 2019 and 2022 took into consideration some possible changes in the initial management of patients following the pandemic.

On another note, our multivariate logistic regression combined clinical relevance and statistical significance during model creation to ensure it is meaningful. Therefore, the variables within the model can be applied in real-world settings. Finally, many variables were integrated in this study to maximize identification of the variables with significant associations with mortality, which strengthens our findings.

## Conclusions

This retrospective study identified significant 28-day mortality predictors among ED patients presenting with bacteremia. The significant predictors included in our multivariate model have not only identified high-risk patients who would benefit from additional targeted care to lower mortality, but also favor an early ID consultation. Although past studies have shown that delayed first antibiotic dose administration and initial inadequate antibiotic increase mortality, our evaluation did not identify these as mortality predictors. However, we do acknowledge our inability to evaluate bacteremia severity scores, which may hinder our ability to draw definitive conclusions regarding the impact of antibiotic timing on 28-day mortality.

An ID consultation for ED patients presenting with bacteremia in general, not restricted to a specific microorganism, was the only modifiable strategy to decrease 28-day mortality with long-term effect. More prospective studies are needed in the future to better understand the impact of this measure depending on the bacteria and on the underlying primary source of infection.

## Data Availability

The datasets used and/or analysed during the current study are available from the corresponding author on reasonable request.

## Abbreviations

CEMI: Centre of Excellence in Infectious Diseases
CHF: Congestive heart failure
CI: Confidence interval
ED: Emergency department
ICU: Intensive care unit
ID: Infectious disease
IV: Intravenous
MI: Myocardial infarction
MSSA: Methicillin-sensitive *Staphylococcus aureus*
OR: Odds ratio
UTI: Urinary tract infection
